# Tear Metabolomics in Dry Eye Disease: A Review

**DOI:** 10.3390/ijms20153755

**Published:** 2019-08-01

**Authors:** Mazyar Yazdani, Katja Benedikte Prestø Elgstøen, Helge Rootwelt, Aboulghassem Shahdadfar, Øygunn Aass Utheim, Tor Paaske Utheim

**Affiliations:** 1 Department of Medical Biochemistry, Oslo University Hospital, Ullevål, 0450 Oslo, Norway; 2 Center for Eye Research, Department of Ophthalmology, Oslo University Hospital, Ullevål, 0450 Oslo, Norway; 3 The Norwegian Dry Eye Clinic, 0366 Oslo, Norway; 4 Department of Medical Biochemistry, Oslo University Hospital, 0027 Oslo, Norway; 5 Department of Plastic and Reconstructive Surgery, Oslo University Hospital, 0450 Oslo, Norway; 6 Department of Maxillofacial Surgery, Oslo University Hospital, 0450 Oslo, Norway; 7 Department of Ophthalmology, Vestre Viken Hospital Trust, 3019 Drammen, Norway; 8 Department of Ophthalmology, Stavanger University Hospital, 4011 Stavanger, Norway; 9 Department of Clinical Medicine, Faculty of Medicine, University of Bergen, 5020 Bergen, Norway; 10 Department of Ophthalmology, Sørlandet Hospital Arendal, 4604 Arendal, Norway; 11 Department of Life Sciences and Health, Oslo Metropolitan University, 0130 Oslo, Norway; 12 Department of Computer Sciences, Oslo Metropolitan University, 0130 Oslo, Norway; 13 Department of Product Design, Oslo Metropolitan University, 0130 Oslo, Norway

**Keywords:** metabolomics, tear, dry eye disease, ophthalmology

## Abstract

Dry eye disease (DED) is a multifactorial syndrome that can be caused by alteration in the quality or quantity of the precorneal tear film. It is considered one of the most common ocular conditions leading patients to seek eye care. The current method for diagnostic evaluations and follow-up examinations of DED is a combination of clinical signs and symptoms determined by clinical tests and questionnaires, respectively. The application of powerful omics technologies has opened new avenues toward analysis of subjects in health and disease. Metabolomics is a new emerging and complementary research discipline to all modern omics in the comprehensive analysis of biological systems. The identification of distinct metabolites and integrated metabolic profiles in patients can potentially inform clinicians at an early stage or during monitoring of disease progression, enhancing diagnosis, prognosis, and the choice of therapy. In ophthalmology, metabolomics has gained considerable attention over the past decade but very limited such studies have been reported on DED. This paper aims to review the application of tear metabolomics in DED.

## 1. Introduction

**Figure 1:**
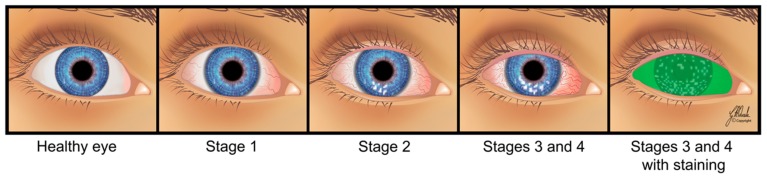
Four stages of dry eye disease (DED) (from left to right): mild, temporary ocular discomfort; chronic and severe pain with deterioration of visual function. Severe DED (Stages 3 and 4) with bulbar redness; some filaments and multiple corneal wounds (stained with fluorescein).

Tears are necessary for maintaining the health of the ocular surface and normal vision. Several internal and external factors may affect tear film composition, integrity, and stability negatively, resulting in dry eye disease (DED). It is considered one of the most common ocular conditions leading patients to seek eye care. Although DED may be asymptomatic, symptomatic patients often report a gritty/foreign body sensation in the eye, discomfort, dryness, photophobia, irritation, burning, stinging, pain, and blurred vision. The stages of DED can range from mild, temporary ocular discomfort to chronic, severe pain with deterioration of visual function (Figure 1). It imposes considerable economic and medical resource usage as well as quality-of-life burden on patients and society [1,2,3,4,5,6,7,8,9,10,11].

Historically, dry eye as a symptom was first observed in 1925 by the French dermatologist Henri Gougerot as part of a generalized condition along with dry mouth, larynx, nose, and vulva [12]. It was later described by the Swedish ophthalmologist Henrik S.C. Sjögren in his 1933 doctoral thesis entitled “*A general disease which attacks chiefly the eyes as well as the lacrimal and salivary glands*”. He named the condition of 19 female patients with arthritis associated with lacrimal dysfunction and dryness as keratoconjunctivitis sicca, meaning “inflammation of the cornea and conjunctiva” [13]. Later, the term “dry eye” was reintroduced in 1950 by Andrew De Roetth, followed by W. Morgan’s 1954 publication, drawing attention internationally [14,15,16,17]. In addition, alternative terms, such as dry eye syndrome, dysfunctional tear syndrome, and lacrimal keratoconjunctivitis have been used in the literature [18].

In the past two decades, several attempts have been made to update the DED definition based on collective experience and knowledge. It was formally defined in 1995 by the National Eye Institute/Industry working group on clinical trials in dry eye [19] followed by the Tear Film and Ocular Surface Society Dry Eye Workshop in 2007 (TFOS DEWS I) [20]. Finally, TFOS DEWS II in 2017 defined DED as a multifactorial disease characterized by loss of homeostasis, in which tear film instability and hyperosmolarity, ocular surface inflammation and damage, and neurosensory abnormalities play etiological roles in the vicious cycle of DED [21]. An alternative definition was put forward by the Asian Dry Eye Society: “Dry eye is a multifactorial disease characterized by unstable tear film causing a variety of symptoms and/or visual impairment, potentially accompanied by ocular surface damage” [22].

**Figure 2:**
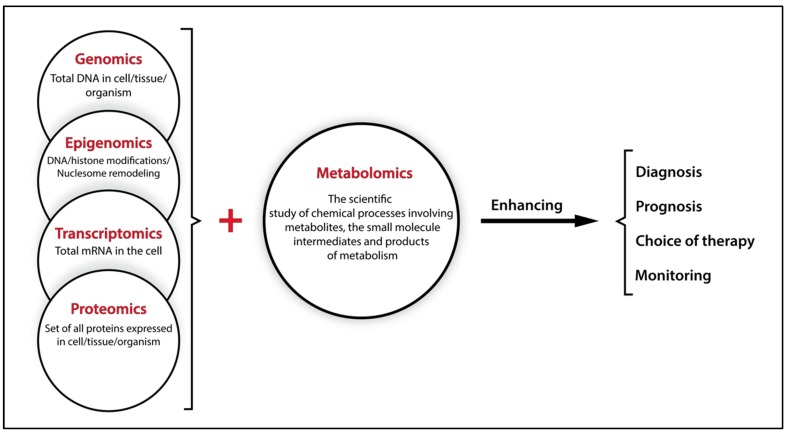
The interaction between the different layers of a biological system and the central role of metabolites in projecting molecular information as they are the end products of gene, mRNA, and protein activity. The identification of distinct metabolites in patients enhances diagnosis, prognosis, the choice of therapy, and monitoring.

Improved DED definition, classification, diagnosis, and treatment have always relied on scientific and technological advances. With the gradual shift from classic hypothesis-driven research approaches to hypothesis-generating research approaches using omics technologies, researchers can now analyze the whole genome, transcriptome, proteome, and so on in a particular disease state. Such an advantageous approach prevents bias in the search for biological mechanisms by prior knowledge and provides deeper understanding of disease processes at the molecular level [23]. However, single omics cannot explain the complex interactions of genes, RNA, proteins, and environmental factors (collectively forming the phenotype) in biological processes in health and disease. The central role of metabolites in projecting molecular information makes them attractive candidates as they are dynamic indicators of gene, mRNA, and protein function in the prevailing internal and external environment (Figure 2). Therefore, metabolomics is potentially a powerful tool for unraveling these complexities and providing insights into the etiology, progression, and therapeutic responses of disease [24].

Metabolomics is a new emerging and complementary research discipline to all omics. It is defined as a comprehensive analysis of metabolites in a biological system and changes in the quantity of metabolites or fluxes (rate of molecule turnover) driven by genetics, (patho)physiological stimuli and/or environmental perturbations [25,26]. Metabolites are generally intermediates and products of cellular metabolism with sizes typically <1 kDa ranging from the most water soluble to the highly lipophilic. Sample preparation procedures and methodological setup define which part of the metabolome can be studied. Traditionally, the main concern of metabolomics has been water-soluble metabolites. Analysis of the most lipophilic part of the metabolome requires dedicated sample preparation, analysis set up, and interpretation software, and is often referred to as lipidomics [27,28,29,30].

Any changes in the normal function of a tissue and/or an organ are instantly reflected in the concentrations of a whole range of specific metabolites. Comparison of the metabolomic profiles from patients with that of healthy subjects provides valuable information for biomedical and clinical experts [31]. It is important to prevent and correct for sources of bias such as subject recruitment criteria (e.g., DED screening, number of participants, age, diet, lifestyle, and sex), time (morning or afternoon), method of tear sampling (e.g., Schirmer test strip and microcapillary tube), and other pre-analytical factors such as sample handling and storage (storage temperature, freeze–thaw cycles, etc.). These factors can alter the composition of the sample, making the results difficult to interpret [25,32,33,34,35,36,37].

Since its initial applications [38,39,40], metabolomics has been widely used in different types and fields of study [41]. In ophthalmology, metabolomics has gained considerable attention in the past decade (for review see: Mendrick and Schnackenberg [42], Midelfart [43], Tan et al. [24], Lauwen et al. [44], and Chen et al. [45]). Nevertheless, it has remained relatively rare in DED studies. The purpose of the present paper is to review the application of tear metabolomics in DED.

## 2. Dry Eye Disease

### 2.1. Risk Factors and Classification

**Figure 3:**
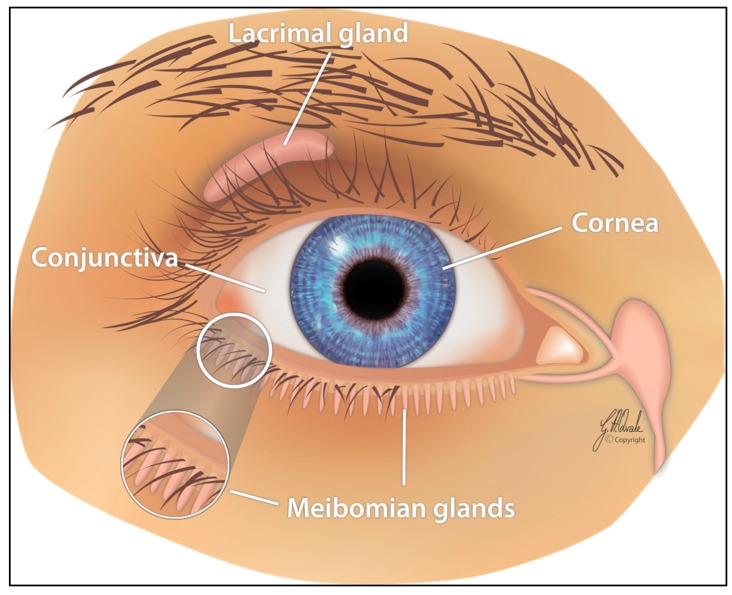
The lacrimal functional unit, responsible for tear secretion, is composed of the ocular surface, main lacrimal gland, and interconnecting innervation.

The ocular surface, an interface between the eye and the environment, consists of the cornea, bulbar and palpebral conjunctiva, and the lacrimal and meibomian glands. The lacrimal functional unit, responsible for aqueous tear secretion, is composed of the ocular surface, main lacrimal gland, and interconnecting innervation (Figure 3). Continuous secretion of tears is stimulated parasympathetically through the nervus intermedius of the nervus facialis, leading to reflectory blinking (approximately 12 blinks per min) with even physiological moistening of the ocular surface in the absence of any irritation. The dysfunction of any component of the lacrimal functional unit can alter the quality or quantity of tears, resulting in DED [46,47,48,49,50,51,52].

The factors in DED formation can be categorized as: (1) Primary factors, e.g., lacrimal or meibomian gland dysfunction or atrophy; (2) secondary factors, e.g., pathological changes in the eyelids, conjunctiva, or cornea; (3) influencing factors, e.g., immunological processes, neurotransmitters, hormones, pharmaceuticals, contact lenses, and environmental pollution; and (4) risk factors contributing to the development of the disease such as sex (i.e., greater risk in female subjects), increasing age, various therapies (e.g., postmenopausal estrogen and radiation), dietary deficiencies (e.g., vitamin A and omega-3 fatty acids), and systemic diseases (e.g., hepatitis C) [46,53,54,55,56,57,58,59,60,61,62,63,64,65,66].

There are two different ways for classification of DED. The TFOS DEWS II report presents a clinically relevant classification, whereas the Madrid triple classification is based on etiology, anatomy pathology, and clinical severity [67,68]. Metabolomics has the potential to generate disease-specific metabolite signatures. Therefore, it can play important roles in aiding DED early diagnosis and determination of underlying etiology and pathology, thereby assisting better estimation of prognosis and facilitating personalized treatment decisions and follow-up monitoring of progression and treatment response.

According to the TFOS DEWS II report, two major divisions are categorized as: (1) Aqueous-deficient dry eye (ADDE), referring to failure of lacrimal secretion and (2) evaporative dry eye (EDE), referring to excessive tear loss from the ocular surface. The former is predominantly caused by Sjögren and non-Sjögren lacrimal disease, whereas the latter results mostly from meibomian gland dysfunction. However, many present with mixed forms of DED [69,70,71].

### 2.2. Tear and Diagnostic Tests

**Figure 4:**
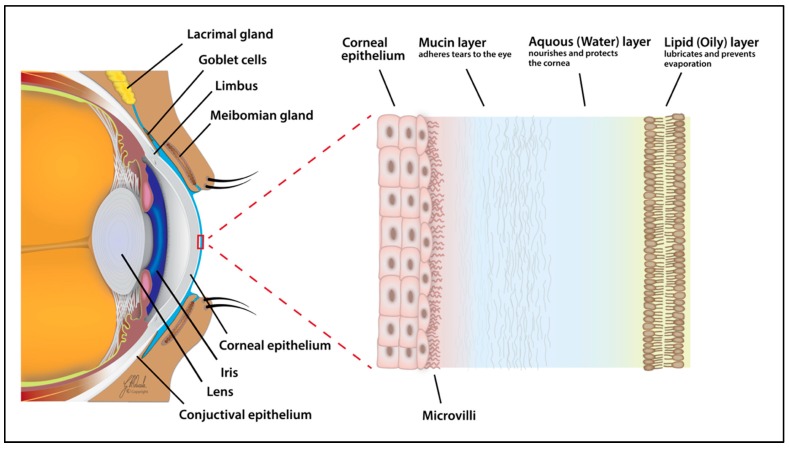
The three-layered structure of the tear film consists of an outer lipid layer at the air surface, an intermediate aqueous layer, and an inner mucus layer on the epithelial surface.

The ocular surface is lubricated, nourished, and protected by tears. This transparent fluid is mostly composed of water (approximately 98%), and 500 solute proteins and electrolytes (approximately 2%) [72]. According to the mode of production, tears are classified into four types: Basal, closed eye, emotional, and reflex [73]. The basal rate of tear secretion is approximately 0.5–2.2 µL/min [74], providing a total volume of 300–400 mL daily [75]. Irritation can increase secretion by up to ~100-fold, reaching a rate of ~300 µL/min [76]. Tear film thickness, measured by different methods, has reported values of ~3–40 µm [77,78]. The tear film structure and composition was first studied in 1946 by Wolff using a slit lamp [79]. Its three-layered structure was defined then as: (1) An outer lipid layer at the air surface; (2) an intermediate aqueous layer; and (3) an inner mucus layer on the epithelial surface (Figure 4). The last two are, however, thought to fall into a single category termed the mucoaqueous layer. Nevertheless, each layer of the tear film cannot be viewed as a distinct temporal entity, particularly for physical properties ensuring the stability of film, but rather as an interlocked part of one unit [73,80].

The meibomian glands produce the surface lipid layer, which reduces evaporation of the aqueous layer and may elastically stabilize the air/tear surface. The surface lipid layer is believed to be a duplex film, which acts as a surfactant and consists of an outer hydrophilic surface on each side of the film and an inner hydrophobic layer of fatty acids. The lacrimal glands produce the middle lubricating aqueous layer, which provides nutrients, suitable osmolarity, and antimicrobial proteins. The goblet cells produce the inner mucus layer, containing high-molecular-weight glycoproteins responsible for ocular surface coating and lowering epithelial cell hydrophobicity [32,73,80,81,82,83,84,85,86,87,88].

To evaluate a subject’s health and pathology in clinical trials effectively, samples should be collected from target tissue rather than the commonly sampled blood or urine. In the eye, collecting tear samples is safer and more convenient for the patient and easier for the ophthalmologists/eye care practitioners compared to samples of conjunctiva and aqueous and vitreous humors. In addition, the complex composition of a small volume of tears gives a good reflection of ocular surface health: Water, inorganic salts (e.g., Na^+^, K^+^, Cl^−^, and Ca^2+^), carbohydrates (e.g., *N*-acetylneuraminic acid and mucins), lipids (e.g., triglycerides, cholesterol, and monounsaturated fatty acids), proteins (e.g., lysozyme, lactoferrin, lipocalin, and immunoglobulin A) and countless metabolites of all kinds [32,86,89,90,91,92].

Several tear-associated clinical tests have been developed for diagnostic evaluations, clinical trials and follow-up examinations of DED. They include the Schirmer test, tear osmolarity, tear film fluorescein break-up time and non-invasive break-up time, ocular surface staining, tear meniscus height, topography and interferometry, aberrometry, and imaging techniques such as meibography and confocal microscopy, and visual function tests. As it is a multifactorial entity, no single tests or cutoff values are pathognomonic to DED. Therefore, clinical tests measuring the signs together with questionnaires that record patient symptoms are currently used [2,7,34,93,94,95,96,97,98,99,100]. These methods have been shown to be associated with the metabolomics approach. The interview, questionnaire, and/or ophthalmologic examinations have previously been used individually, or in combination, to screen and group DED patients for metabolomics studies of tears. Using this approach, distinct metabolomic fingerprints of DED patient and control groups were found [101,102,103,104,105,106,107] and additional severity-associated subgroups, mild-to-moderate and moderate, were identified in the DED patients [103]. Thus, there is considerable potential for metabolomics in the classification of DED.

## 3. Tear Sampling and Analysis

Advances in analytical technologies and instrumentation have had a remarkable effect on human health over the past few decades. In the field of metabolomics, it is now possible to determine the chemical structures of compounds as well as to examine the biochemical processes of organs such as the eye. In order to develop metabolomic tests that can be used in the clinic to diagnose DED, the study design has to be considered carefully. Obtaining good results requires the minimization of potential errors within the total testing process [108,109]. Differences in subject recruitment, tear sampling, and storage can result in variation [25,32,33,34].

Firstly, metabolomics tests must be implemented in large-scale, cross-sectional, and clinical epidemiological studies of DED. As mentioned in Section 2.2, the current method of diagnosing DED is a combination of signs and symptoms using clinical tests and questionnaires [95,100]. The number of participants is essential for increasing the statistical robustness. For example, three-step investigations requiring more than 1000 individuals have been recommended for metabolic profiling studies: (1) Discovery and (2) validation studies as pilots with 20–100+ subjects per class from two independent populations and (3) cohort validation with 1000s of samples [25]. Therefore, researchers should be careful when designing their studies to ensure a sufficient number of subjects is included for making valid conclusions.

Secondly, a feasible technique of tear sampling needs to be implemented. Tear sampling is performed by direct (e.g., microcapillary tube and micropipette) and indirect (e.g., Schirmer test strip, filter paper disk, cellulose sponge, and polyester rod) methods. Among these, the most frequently used are the Schirmer test strip and the microcapillary tube [74,110]. The Schirmer test strip was first introduced by the German ophthalmologist Otto Schirmer in 1903 as a piece of striped and marked (35 × 5 mm) blotting paper installed on the lower eyelid margin with or without anesthesia to collect tears [111,112]. The microcapillary tube is a glass or plastic cylinder with a hollow space inside for collecting tears by holding it horizontally close to the conjunctival sac [48]. The advantage of applying one method over the other has been debated in the literature. The Schirmer paper strip has several drawbacks such as damage to the surface cells of the conjunctiva, irritation, and vascular fragility at the site of its installation; hyperosmolarity of solutes following water evaporation [113]; and/or lower concentration of components owing to potential increased tear flow due to stimulation [114]. However, the method is considered easy, fast, accurate, and simple for analysis [115] and can be used for diagnostic (e.g., DED) as well as molecular biological characterization purposes. It also provides additional time to ophthalmologists/eye care practitioners for recording the patient’s medical history or consulting about possible treatment once the strip is in place [48]. In comparison with the microcapillary tube, the average protein concentration in a tear sample collected by the Schirmer test strip is higher [116,117]. This method is also less invasive [118] and does not require aggressive extraction [115].

Basal and reflex tears are the most common sampling types. A basal tear is the small quantity produced continuously to cover the corneal surface. It is necessary for ocular surface health and is deficient in syndromes such as DED. A reflex tear is produced following stimulation of the reflex arc, e.g., stimulation of the nasal mucosa by the chem-sneeze reflex. Its application is encouraged when the volume of the tear sample is low (e.g., DED), frequent sampling is required, or when the sampling method requires stimulation in advance. For example, the microcapillary tube method often requires pre-stimulation or the instillation of saline at different volumes (100–200 µL) into the cul-de-sac. However, such manipulation can dilute the tear sample, leading to composition change and/or the detection of fewer metabolites [32,103,110,115,119].

Once the tear sample is collected, it is critical to quench the metabolic activity quickly. Otherwise, metabolite turnover (metabolic flux) occurs, rendering the fluid chemically unstable and less representative of the in vivo status. Quenching of ex vivo metabolic activity can be done by an immediate temperature drop to <0 °C followed by storage at −80 °C, or enzyme denaturation by temperature increase or by applying organic solvents. The next step involves extraction of the metabolites of interest present in the complex biological sample by appropriate extraction procedures. Several techniques based on the type of sample, experimental strategy (i.e., targeted and untargeted), and analytical instrument are currently in use [24,25,109].

The analytical step of metabolomic studies is now based on advanced spectroscopy technologies to present complex information. The two main platforms are mass spectrometry (MS) and nuclear magnetic resonance (NMR). MS measures the mass of a molecule by determining the mass-to-charge ratio (m/z) of its ions. For increased sensitivity and better identification, it is usually coupled with separation techniques such as high-performance liquid chromatography (HPLC–MS), gas chromatography (GC–MS), and capillary electrophoresis (CE–MS). NMR measures the magnetic properties of certain atomic nuclei (e.g., ^1^H, ^31^P, and ^13^C) in the metabolites [24,43,103,120]. In comparison with MS, NMR requires minimal sample preparation; provides information on both structure and composition; and is nondestructive, rapid, and low-cost, with high reproducibility over time and unbiased detection. However, MS is more sensitive and can cover a wider range of metabolites [121,122,123,124]. These two platforms can be used at the same time to detect 100s–1000s of metabolites in a process termed metabolic phenotyping or metabotyping [125].

## 4. Metabolomic Studies of Tears in DED

Metabolomics determines the amounts of a wide range of structurally diverse endogenous and exogenous components in a single sample and describes patterns and ratios, yielding information on metabolic pathways and their interactions. The identification of distinct metabolites, selected ratios, or multicomponent patterns in patients can potentially inform clinicians at an early stage or during monitoring of disease progression, enhancing diagnosis, prognosis, and the choice of therapy [31]. This profiling approach has previously been reported in human eye tissues such as the cornea, ciliary body, lens, iris, retina, and aqueous and vitreous humors [126,127,128,129,130,131,132]. Some studies have identified different metabolites in tears for various purposes [118,133,134,135,136,137,138,139], but the application of metabolomics in DED remains very limited, as described chronologically below. Table 1 presents the methodological details and main metabolite findings in these studies.

First, Peral et al. [101] analyzed the levels of the diadenosine polyphosphates, Ap_4_A and Ap_5_A, in tear samples from patients with DED, both symptomatic and asymptomatic, and from control subjects using HPLC. The symptomatic DED group had higher levels of these dinucleotides, particularly subjects with low tear secretion, than the controls. Additionally, there was a significant sex-specific difference in the Ap_4_A and Ap_5_A levels of the symptomatic subjects.

Regardless of tear secretion levels, asymptomatic and symptomatic women showed higher concentrations than symptomatic men. Due to low sample volumes, analysis was not possible for subjects with very low tear secretion, which in the study encompassed female participants only. Despite the possible selection bias toward less severe DED in the female group, the sex differences for Ap_4_A and Ap_5_A were significant (*p* < 0.05). The study suggested these two substances, particularly Ap_4_A, as objective biomarkers of DED. Diadenosine polyphosphates are naturally occurring compounds, which were first described in the tear film by Pintor et al. [141]. Although their roles and mechanisms of action are not fully understood, they were shown to modulate intraocular pressure as well as to accelerate corneal wound healing by acting through P_2_ receptors [142,143]. The release of diadenosine polyphosphates from ocular epithelial cells into the tear film has been proposed to act through ATP binding cassette transporter, the cystic fibrosis transmembrane conductance regulator, or glycoprotein P [144]. The association between these proteins and DED would be interesting to investigate in future. 

Thereafter, Pescosolido et al. [102] investigated the presence of carnitine and its derivatives, l-acetylcarnitine and l-propionylcarnitine, in tears and compared their levels in DED and control subjects to shed light on the potential role of carnitine as an osmoregulatory, antioxidant, and antiapoptotic agent. Their analysis, using HPLC–MS, confirmed the presence of significantly lower concentrations of carnitine, l-acetylcarnitine, and l-propionylcarnitine in the tear fluids of patients with DED than in the controls (*p* < 0.05). Therefore, the authors proposed a possible protective effect of carnitine by preventing the damaging impacts of a hypertonic tear film. The protective role of carnitine is thought to be due to potentiation of two free-radical scavenging enzymes’ activity in tear film—catalase and glutathione peroxidase [145]. The presence of low levels of carnitine in the tear film was linked to active transport across the cell membrane of ocular tissues [146,147]. The link between transporters and DED is an intriguing topic, which should be explored in further research.

Later, Galbis-Estrada et al. [103] examined the metabolomic profile of reflex tears from DED and healthy groups using proton NMR spectroscopy (^1^H-NMR). Additionally, the authors considered two severity-associated subgroups, i.e., mild-to-moderate and moderate, in that study. They found intergroup differences mainly in the levels of cholesterol, *N*-acetylglucosamine, glutamate, creatine, amino-n-butyrate, choline, acetylcholine, arginine, phosphoethanolamine, glucose, and phenylalanine. In addition, their metabolic discrimination model enabled the identification of two subgroups. The most important metabolites for intragroup comparison were: –CH3 lipids, cholesterol/lipids, *N*-acetylglucosamine, glutamate, amino-n-butyrate, choline, glucose, phenylalanine, and formate. Then, Galbis-Estrada et al. [104] extended their investigation by prescribing to participants an oral nutraceutical supplement (containing antioxidants and essential polyunsaturated fatty acids; Table 1) of three capsules a day for three months. The DED and control groups showed different basal tear metabolomic profiles and the authors could identify ~50 metabolites of cholesterol, *N*-acetylglucosamine, glutamate, amino-n-butyrate, choline, glucose, and formate before supplementation and of choline/acetylcholine after supplementation. The authors concluded that the tear metabolomic profile of patients with DED can be modified with appropriate oral supplementation containing antioxidants and essential fatty acids. This finding demonstrates the potential influence of diet and oral medication on tear composition and DED. Next, Pieragostino et al. [105] aimed to develop and apply a robust, specific, and selective method for measuring steroid levels using HPLC–MS in tear samples and for testing their diagnostic power. They simultaneously quantified cortisol, corticosterone, 11-deoxycortisol, 4-androstene-3,17-dione, testosterone, 17α-hydroxyprogesterone, and progesterone with good predictive power enabling identification of patients with DED. The study suggested, for the first time, a new approach for investigating steroid profiling directly in tear fluids. The strong association between the levels of serum estrogen and development/progression of DED has been previously shown [148]. In this respect, the potential of manifested DED in women during hormonal fluctuation (e.g., taking oral contraceptive, pregnancy, lactation, and post menopause) is well known [149,150]. Regarding the tear film, the normal function of lacrimal gland and its structural organization have been linked to androgen levels. In addition, optimal tear production requires an adequate hormonal environment, provided by prolactin and estrogens [151]. The identification of receptors of androgens, estrogens, progesterone, and prolactin in several ocular tissues (e.g., main lacrimal gland and MGs) has provided additional proof of sex-hormone involvement in the pathophysiology of different ocular surface diseases including DED. 

English et al. [140] tested specialized pro-resolving mediators (SPMs), actively involved in reducing ocular inflammation, in emotional tear samples from healthy subjects. By using HPLC–MS, the authors identified pro-inflammatory (prostaglandins and leukotriene B4) and pro-resolving lipid mediators (D-series resolvins, protectin D1, and lipoxin A4), which did not include the maresin family (maresin 1 and 2). Furthermore, sex-specific differences were observed through the lack of SPMs (RvD1, RvD2, RvD5, and PD1) production in female subjects. The SPM is composed of four mediator families: Resolvins, lipoxins, protectins, and maresins [152]. They have been shown to be associated with DED. For example, the resolvins are synthesized from omega-3 (eicosapentaenoic acid and docosahexaenoic acid) and omega-6 (arachidonic acid) fatty acids. The precursors of these fatty acids are present in tears. Their correlations with clinical tests of DED have been documented [153]. Finally, Chen et al. [107] evaluated the metabolomic profile of tears from DED and healthy groups using nanoLC–MS. They identified a total of 156 metabolites, of which 32 were significantly changed (*p* < 0.05) in patients with DED. These DED-specific metabolites belonged to eight superclasses: Benzenoids; hydrocarbons; lipids and lipid-like molecules; nucleosides, nucleotides, and analogs; organic acids and derivatives; organic nitrogen compounds; phenylpropanoids and polyketides; and unknown. The study suggested potential DED biomarkers and revealed that metabolic processes related to glycolysis/gluconeogenesis, amino acid metabolism, and the complement and coagulation cascades were involved in DED.

The experimental workflow of the abovementioned studies can be divided into targeted and untargeted approaches. The former is a hypothesis-testing approach and examines a small number of specific metabolites, providing accurate and precise analysis. In contrast, the latter, also termed metabolic profiling, is performed when there are differences between two study groups (e.g., case and control) but the specific differences are not known. In other words, this approach is applied when a general hypothesis is available or when a researcher intends to generate hypotheses. Targeted studies are usually performed after untargeted studies for validation purposes [24,154]. Targeted analyses of tear samples have long been performed, but a group of scientists in Singapore was the first to establish an analytical platform for the global characterization of metabolites in human tear samples [106]. They could identify 60 metabolites from a wide range of compound classes in healthy subjects using HPLC–MS. The application of this development has been suggested to be used in future untargeted metabolomics of ocular diseases, particularly for DED.

In addition to tear analysis from patients with DED, metabolomics has previously been performed on non-tear samples. For example, Chen et al. [155] evaluated metabolite changes in human conjunctival epithelial cells using metabolomics and reported 21 metabolites from different classes. Among these, glycerophosphocholine was identified as the main osmoprotectant. Vehof et al. [156] investigated the associations of 222 known serum metabolites in DED using HPLC–MS and GC–MS. Their findings indicated significantly decreased levels of androsterone sulfate and epiandrosterone sulfate in DED subjects. The next strongest association was for 4-androsten-3β, 17β-diol disulfate 1 and 2, and dehydroepiandrosterone sulfate. Moreover, decreased glycerophosphocholines were associated with DED.

## 5. Conclusions and Future Work

Metabolomics is a new emerging, promising, and powerful tool for multidimensional analysis of metabolic status and events in biological systems subject to internal and external influences. The identification of single metabolites, metabolite ratios, and distinct metabolomic profiles in patients can potentially be used as biomarkers of DED, aiding earlier and more precise diagnosis, monitoring of disease progression, prognosis evaluation, and the choice and monitoring of therapy. DED research to date has focused on comparing the metabolomic profiles from patients with that of healthy subjects in targeted and untargeted studies. Although this approach has provided better understanding of the metabolic changes associated with disease processes, future studies should also aim at identifying and validating unique biological markers and multicomponent patterns. Tear fluid most certainly contains far more metabolites than so far reported. Continuous improvements in instrumentation, software, and metabolite databases used for metabolomics will therefore reveal new and relevant metabolites. As the number of subjects included in the published reports is relatively low, increasing the number of participants in future studies can also help to improve the power of the results obtained. Additionally, research questions that could be asked include the effect of medication, chemical, radiation, nutritional, and other exogenous (environmental) and endogenous factors on patients with DED. These all require precise phenotypic classification of patients, standardized sampling, sample processing, and improved experimental techniques, instrumentation, data analysis, and results interpretation in the field of metabolomics.

## Figures and Tables

**Table 1:** The methodological details of metabolomic studies in DED.

Subjects	DED Screening	Sampling	Analytical Method	Metabolites (DED vs. Control)	Reference
Total, 97 subjects (27 men, 70 women); mean age, 27 ± 1 years (range, 20–36 years)	McMonnies questionnaire, Schirmer test	Tears collected on Schirmer paper strip, transferred to an Eppendorf tube containing 500 µL ultrapure water, and frozen	HPLC	↑Diadenosine polyphosphates Ap_4_A and Ap_5_A	[101]
Total, 20 subjects (10 healthy, 10 DED); mean age 65 years (range, 55–75 years)	Ophthalmologic examinations	Tears collected in a capillary tube, pooled from both eyes, if required, and stored at 4 °C	HPLC–MS	↓Carnitine and its derivatives l-acetylcarnitine and l-propionylcarnitine	[102]
Total, 90 subjects (35 healthy with median age of 36 ± 11 years; 55 DED with median age of 52 ± 18 years); age, 25–80 years	Interview, OSDI* questionnaire, ophthalmologic examinations	Reflex tears (20–30 μL) collected from both eyes with a microglass pipette, deposited in a cryotube, and stored at −80 °C	^1^H-NMR	−CH3 lipids, cholesterol/lipids, *N*-acetylglucosamine, glutamate, amino-n-butyrate, choline, glucose, phenylalanine, and formate	[103]
Total, 90 subjects (35 healthy, 55 DED); mean age, 52 years (range, 25–80 years)	Interview, OSDI questionnaire, ophthalmologic examinations	Reflex tears (18–35 μL) collected with a microglass pipette, transferred to an Eppendorf tube, and stored at −80 °C	^1^H-NMR	←Cholesterol, *N*-acetylglucosamine, glutamate, amino-n-butyrate, choline, glucose, and formate →choline/acetylcholine	[104] **
Total, 27 female subjects (13 healthy, 14 DED); age, 25–73 years	Schirmer test	Tears collected on Schirmer paper strip, transferred to an Eppendorf tube, dried at room temperature, and stored at −80 °C	HPLC–MS	Cortisol, corticosterone, 11-deoxycortisol, 4-androstene-3,17-dione, testosterone, 17α-hydroxyprogesterone, and progesterone	[105]
Total, 12 healthy subjects (six men, six women)	–	Emotional tears collected and stored at −80 °C	HPLC–MS	N/A	[140]
Total, 37 subjects (19 healthy, 18 DED); (six men, 31 women); age, 18–87 years	Interview, ophthalmologic examinations	Tears (5−15 μL) collected with a capillary tube, transferred to an Eppendorf tube, and stored at −80 °C	nanoLC–MS	Benzenoids; hydrocarbons; lipids and lipid-like molecules; nucleosides, nucleotides, and analogs; organic acids and derivatives; organic nitrogen compounds; phenylpropanoids and polyketides; and unknown	[107]
Total, six healthy subjects	No ocular complaints or history of contact lens usage	Tears collected on Schirmer paper strip and stored at −80 °C	HPLC–MS	N/A	[106]

* OSDI: Ocular surface disease index. ** Components of the capsule given to participants for three months: docosahexaenoic acid (350 mg), eicosapentaenoic acid (42.5 mg), docosapentaenoic acid (30 mg), vitamin A (133 mg), vitamin C (26.7 mg), vitamin E (4 mg), tyrosine (10.8 mg), cysteine (5.83 mg), glutathione (2 mg), zinc (1.6 mg), copper (0.16 mg), manganese (0.33 mg), and selenium (9.17 mg). Arrows: ↑ Increased or ↓ decreased levels of metabolites in pathology DED patients compared to samples from healthy individuals. ← Before or → after supplementation**. N/A: Not applicable
